# A Parallel Population Genomic and Hydrodynamic Approach to Fishery Management of Highly-Dispersive Marine Invertebrates: The Case of the Fijian Black-Lip Pearl Oyster *Pinctada margaritifera*

**DOI:** 10.1371/journal.pone.0161390

**Published:** 2016-08-25

**Authors:** Monal M. Lal, Paul C. Southgate, Dean R. Jerry, Cyprien Bosserelle, Kyall R. Zenger

**Affiliations:** 1 Centre for Sustainable Tropical Fisheries and Aquaculture, James Cook University, Townsville, Queensland, Australia; 2 College of Science and Engineering, James Cook University, Townsville, Queensland, Australia; 3 Australian Centre for Pacific Islands Research, Faculty of Science, Health, Education and Engineering, University of the Sunshine Coast, Maroochydore, Queensland, Australia; 4 Geoscience Division, Secretariat of the Pacific Community, Nabua, Suva, Fiji Islands; University of British Columbia Okanagan, CANADA

## Abstract

Fishery management and conservation of marine species increasingly relies on genetic data to delineate biologically relevant stock boundaries. Unfortunately for high gene flow species which may display low, but statistically significant population structure, there is no clear consensus on the level of differentiation required to resolve distinct stocks. The use of fine-scale neutral and adaptive variation, considered together with environmental data can offer additional insights to this problem. Genome-wide genetic data (4,123 SNPs), together with an independent hydrodynamic particle dispersal model were used to inform farm and fishery management in the Fijian black-lip pearl oyster *Pinctada margaritifera*, where comprehensive fishery management is lacking, and the sustainability of exploitation uncertain. Weak fine-scale patterns of population structure were detected, indicative of broad-scale panmixia among wild oysters, while a hatchery-sourced farmed population exhibited a higher degree of genetic divergence (*F*_st_ = 0.0850–0.102). This hatchery-produced population had also experienced a bottleneck (*N*_*eLD*_ = 5.1; 95% C.I. = [5.1–5.3]); compared to infinite *N*_*eLD*_ estimates for all wild oysters. Simulation of larval transport pathways confirmed the existence of broad-scale mixture by surface ocean currents, correlating well with fine-scale patterns of population structuring. *F*_*st*_ outlier tests failed to detect large numbers of loci supportive of selection, with 2–5 directional outlier SNPs identified (average *F*_st_ = 0.116). The lack of biologically significant population genetic structure, absence of evidence for local adaptation and larval dispersal simulation, all indicate the existence of a single genetic stock of *P*. *margaritifera* in the Fiji Islands. This approach using independent genomic and oceanographic tools has allowed fundamental insights into stock structure in this species, with transferability to other highly-dispersive marine taxa for their conservation and management.

## Introduction

Sustainable management and conservation of marine species is faced with a number of challenges, among which is the wide distribution of taxa across diverse habitats and geopolitical jurisdictions, that make species-specific management plans difficult to design and implement. Many taxa also face high rates of exploitation, that in some cases has led to the collapse or abnormally slow recovery of wild fisheries, bringing into question whether current management strategies are effective or appropriate [1–3]. The need for accurate fishery management has resulted in the development of the stock concept for aquatic species, which can allow for targeted conservation efforts and informed exploitation, once stock boundaries have been defined [2,4]. Despite the usefulness and importance of the stock concept, there is currently no clear consensus on what constitutes a stock, and numerous definitions in the literature shift emphasis for defining stock boundaries between the degree of demographic homogeneity within stocks, and their reproductive isolation [5]. Since a stock is the fundamental unit used for fishery assessment and administration, it is imperative that the spatial boundaries delineated are also biologically meaningful, to ensure correct management action [3,6].

For assessment of a particular stock, it is important to determine the number and extent of populations being examined. However, the biological concept of a population has either ecological (demographic interactions of individuals), or evolutionary (genetic structuring) aspects [3,5]. Reiss et al. [3] make the observation that many fishery management and assessment tools focus primarily on the ecological aspects of populations (e.g. population growth and mortality rates), while overall management goals also include many evolutionary criteria such as the conservation of genetic diversity and maintenance of sustainable spawning stock biomass. This disconnect highlights the need for bridging the gap between fisheries management and population genetics, and particularly for characterising stock boundaries, identifying the level of divergence required to manage two populations together, or as separate entities [3–7].

A major problem posed for application of the stock concept in the marine environment is the relative absence of barriers to dispersal and migration compared to terrestrial systems, and the highly-dispersive larval stages of many species [2]. For species which are either highly mobile and/or broadcast spawners with prolonged pelagic larval duration (PLD), the potential for gene dispersal is high, often resulting in weak population differentiation that is evident over large geographic distances [6,8–10]. Furthermore, despite the presence of weak population structure, selective forces can produce fitness differences between populations through local adaptation [11].

For a large number of species that exhibit high levels of gene flow, low levels of genetic structure may be present, but difficult to detect [2,3]. The importance of detecting low, but biologically significant differentiation for understanding the ecology and evolution of these taxa, and implications for their conservation and management is discussed by André et al. [12], Gaggiotti et al. [7], Hauser and Carvalho [13], Palumbi [9, 14], Waples [2] and Waples and Gaggiotti [6]. It is clear from these studies that a common solution for delimiting population and stock boundaries in high gene flow species is not possible, but rather assessment on an individual basis is required, taking into consideration the biological, ecological and fishery management issues involved. Additionally, in situations where traditional stock assessment is not possible (e.g. due to logistical or financial reasons), genetic approaches examining fine-scale population structure and functional differences (such as local adaptation), can be important for resolving stock boundaries.

A potential solution in recent years has been the use of genome-wide SNPs, which can reveal fine-scale patterns of population structure to highlight differences between populations, and also detect signatures of selection [15–17]; with much higher resolving power than traditional markers (e.g microsatellites and mtDNA). However, while genetic analyses by themselves are a powerful tool for investigating population connectivity and structure, consideration of other data for defining stocks such as phenotypic information, demographic data, or biophysical modelling should not be overlooked [3,18,19]. For broadcast spawning species with prolonged PLD, investigations considering independent environmental and molecular data together, can provide unrivaled insights into the biological and physical processes that organise and regulate population structure [4,20]. Hydrodynamic dispersal modelling is an analysis tool that relies on oceanographic data, and can be used for simulation and independent inference of larval dispersal from source to sink locations [20,21]. Because many marine species produce large quantities of very small larvae with variable PLD that makes tagging and tracking studies very difficult, highly realistic estimates of population connectivity can be achieved when hydrodynamic dispersal data are combined with genetic analyses [4,20,22–24].

Bivalve molluscs present a number of unique challenges for stock assessment, which include highly variable patterns of larval dispersal and recruitment. Additionally, traditional bivalve stock assessment surveys typically require extensive sampling to determine distribution and abundance, which in most situations can be costly and impractical. Because the adults of many taxa are sedentary and recruitment rates highly variable, a stock may occupy a discrete geographic region as large as an entire reef system, or as small as a single bivalve bed [25]. When coupled with the homogenising effects of larval exchange over large distances, accurate stock assessment can quickly become problematic. For many bivalves, and pearl oysters in particular, examination of morphological differences for stock assessment primarily relies on variable shell characters to elucidate differences between individuals, populations and species [26]. This can be a difficult exercise, particularly during early stages of development [27], as factors including phenotypic plasticity and environmental effects can confound measurements. In recent times, molecular methods have been increasingly relied upon to provide solutions to these problems [26,28].

In French Polynesia, the black-lip pearl oyster *Pinctada margaritifera* (Pteriidae) displays substantial genetic fragmentation, despite being a broadcast-spawner with an extended PLD of 26–30 days [29,30]. This has been related primarily to habitat heterogeneity, with significant genetic structure detected between open and closed atoll lagoon systems [31,32]. Here, detection of both fine-scale and broad-scale patterns of differentiation were identified as being biologically important for fishery and aquaculture management [33,34]. For the Fiji Islands, cultured pearl and pearl shell production from *P*. *margaritifera* is a valuable industry and substantial source of coastal community livelihoods. It produces a high-value, low-volume and non-perishable product with a comparatively smaller environmental footprint than most other forms of aquaculture, making it an ideal export commodity for developing Pacific island countries [35–37]. The industry is almost exclusively dependent on wild oysters for which there are currently no comprehensive fishery management guidelines, and therefore no information is available on the number of discrete populations present, their levels of genetic fitness and relatedness, or if domestic translocation of animals is suitable for the establishment of new pearl farms.

Two preliminary stock assessment surveys using traditional methods reported low abundances of *P*. *margaritifera* at all locations examined, and recommended immediate conservation efforts to increase population densities of wild oysters [38,39]. A previous study which examined oysters sampled at four Fijian sites discovered a mixed pattern of population structure, and identified a need for comprehensive evaluation of additional populations to determine country-wide patterns of genetic structure and connectivity [17]. In this study, we assess the stock structure of *P*. *margaritifera* in the Fiji Islands for fishery and aquaculture management, using independent population genomic and hydrodynamic modelling approaches. This work provides valuable insights for the fishery management and aquaculture of this commercially important bivalve mollusc, and also demonstrates solutions for challenges that apply to stock assessment efforts in other broadcast-spawning marine taxa, that possess similar life history characteristics.

## Methods and Materials

### Specimen collection, tissue sampling and DNA extraction

**Fig 1:**
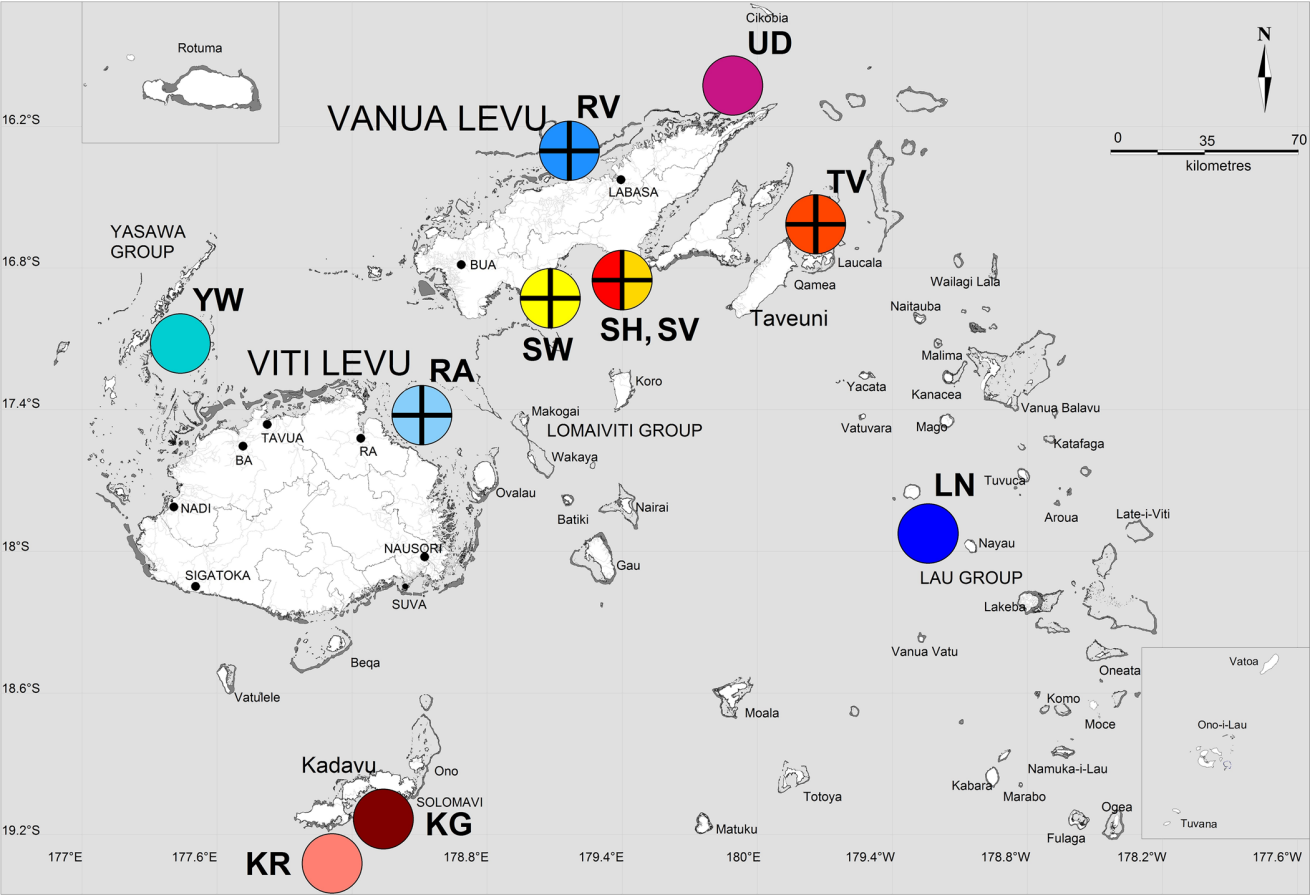
Map of sampling locations in the Fiji Islands adapted from Lal et al. [17], where wild and farmed *P*. *margaritifera* were collected. Reef outlines are presented in dark grey, and site colours correspond to population colour codes used for Figs 2 and 3. Solid circles represent wild oyster collection sites, while circles superimposed with a cross indicate farm locations. Site codes represent the following locations: YW, Naviti Island in the Yasawa group; RA, farm site at Namarai, Ra; SW, farm site at Wailevu, Savusavu; SH, farm site at Wailevu, Savusavu for hatchery produced oysters; SV, farm site at Vatubukulaca, Savusavu; RV, farm site at Raviravi; UD, Vunikodi, Udu Point; TV, farm site at Wailoa, Taveuni; LN, Nayau Island in the Lau group; KG, Galoa Island off Kadavu Island and KR, Ravitaki on Kadavu Island.

Adult and juvenile *P*. *margaritifera* (n = 427) sized between 7–18 cm in dorso-ventral measurement (DVM), were collected from 11 sites in the Fiji Islands representing both farmed and wild populations country-wide, from December 2012 to October 2013 (Fig 1). Permission to sample wild sites was obtained from Fijian traditional fishing ground (*i qoliqoli*) custodians, while farm site access was permitted by farm owners. The vast majority of farmed oysters are collected as settling wild juveniles or spat, that recruit to dedicated settlement substrates deployed by farms. Additionally, limited numbers of individuals are propagated in a single hatchery, and are the progeny of wild-sourced broodstock. Oysters are grown in pocket panel nets that are suspended in the water column from long lines [40]. At all farm sites, wild populations are present in adjacent habitats. Farmed oysters were sampled at Ra (n = 50), Raviravi (n = 32), Taveuni (n = 43) and three locations in Savusavu: Vatubukulaca (n = 50); Wailevu (n = 49) and a hatchery-produced population also at Wailevu (n = 50). Oysters collected from all farms originated either from spat collectors [40], or were gleaned from adjacent coral reef habitats. Wild populations were sampled at two sites on the Island of Kadavu (Galoa Island; n = 25 and Ravitaki; n = 25), the Yasawa archipelago (Naviti Island; n = 35), Udu Point (n = 18) and the Lau archipelago (Nayau Island, n = 50). Two sites were sampled on Kadavu to detect any differentiation present between adjacent locations due to environmental heterogeneity (e.g. reef effects). Proximal mantle and adductor muscle tissues (1.5 and 1 cm respectively), were removed and transferred to tubes containing 20% salt saturated dimethyl sulfoxide (DMSO-salt) preservative [41]. All oysters were handled in accordance with James Cook University's animal ethics requirements and guidelines. Genomic DNA was extracted using a modified cetyl trimethyl ammonium bromide (CTAB, Amresco, cat. #0833-500G) chloroform/isoamyl alcohol protocol, with a warm (30°C) isopropanol precipitation [42]. To clean up all DNA extractions, a Sephadex G50 [43] spin column protocol was used, prior to quantification with a Nanodrop 1000 Spectrophotometer (Thermo Scientific).

### ddRADseq library preparation and sequencing

Double digest restriction site-associated (ddRAD) libraries were prepared following the methods of Peterson et al. [44], with a number of modifications for *P*. *margaritifera* as described by Lal et al. [17]. Briefly, nine libraries in total were prepared (48 barcoded individuals per pool × nine unique Illumina TruSeq indices), from which four libraries were pooled at equimolar ratios for sequencing in one lane, while the remaining five libraries were pooled for a second lane. After cluster generation and amplification (HiSeq PE Cluster Kit V4 cBOT), 100 bp paired-end sequencing was performed on an Illumina HiSeq 2000 platform at the Australian Genome Research Facility (AGRF) in Melbourne, Victoria.

### Sequence quality control, marker filtering and genotype calling

Raw reads obtained following sequencing were processed as described by Lal et al. [17], with all read filtering and SNP genotyping carried out using STACKs v.1.20 software [45,46]. From all available SNPs, only the most informative SNP per locus was selected for further analysis, as per Lal et al. [17]. Final genotypes were called at a Minor Allele Frequency (MAF) of 2% and minimum stack depth of 10, with the minimum proportions of loci allowed across individuals set at 20%, and across populations at 50% (-r and -p options respectively). In addition, each unique SNP was genotyped in at least 10 individuals within a population, and represented in a minimum of two populations across the whole dataset [47].

All loci were screened using Arlequin v.3.5.1.3 [48] for departure from Hardy-Weinberg Equilibrium (HWE), and removed if deviations were significant after FDR correction (p<0.00001), or loci were monomorphic across all populations [49,50]. All loci were also tested for genotypic linkage disequilibrium (LD) in Genepop v.4.3 [51], as per Lal et al. [17]. Additionally, all loci were compared with NCBI viral and bacterial sequence databases using Basic Local Alignment Search Tool (BLAST) searches [52], to detect contamination which may have occurred during library preparation, with all matching loci excluded from the final dataset.

### Evaluation of genetic diversity, inbreeding and population differentiation

For assessment of genetic diversity within and between populations, allelic diversity indices including average observed (*H*_o_), and average expected heterozygosities corrected for population sample size (*H*_n.b._) were computed. Inbreeding coefficient (*F*_is_) calculation and estimation of the effective population size based on the linkage disequilibrium method (*N*_*eLD*_) was also carried out for each population, all using Genetix v.4.05.2 [53] and NeEstimator v.2.01 [54]. Furthermore, family relationships among all individuals within sampled populations were assessed with ML-RELATE [55], which allowed for the identification of any parent-offspring, full-sib or half-sib pairs present. Relationships between individuals from different regions were also evaluated by assessing all populations together, in order to detect migration.

High levels of genome-wide polymorphism characterise many bivalves and other marine invertebrates, which may affect RADseq-based genotyping approaches by disproportionately sampling the genome due to mutations in restriction enzyme cut sites [56,57]. As previously outlined by Lal et al. [17] for *P*. *margaritifera*, to ascertain the potential degree of bias, *F*_is_ and heterozygosity were calculated for the dataset during preliminary testing at a range of missing data thresholds from 80 to 20%. These parameters were also calculated at varying read depths per stack from 5 to 15 (in the STACKs 'populations' module), before performing final *F*_is_ and heterozygosity computations. Heterozygosity and *F*_is_ changed with increasing read depth per stack from 3 to 6, however, no substantial change occurred beyond a read depth of 7. Based on these results, a final read depth threshold of 10 was selected for generating final genotypes.

To investigate individual genomic levels of diversity, multi-locus heterozygosity was examined, with the standardised heterozygosity (SH) and internal relatedness (IR) computed for each population with the *R* package *Rhh* [58,59]. Furthermore, the average multi-locus heterozygosity (Av. MLH) per population was computed manually following Slate et al. [60], along with the proportion of rare alleles with a MAF <5%. To investigate levels of population structure between sampling locations, pairwise *F*_st_ estimates for each population were calculated using Arlequin v.3.5.1 [48] with 10,000 permutations, and broad-scale population structure visualised by performing a Discriminant Analysis of Principal Components (DAPC) in the *R* package *adegenet* 1.4.2 [59,61–63]. The DAPC was carried out for all loci, and an α-score optimisation used to determine the number of principal components to retain. Additionally, the ‘find.clusters’ function of *adegenet* was utilised to determine the optimal number of actual clusters using the Bayesian Information Criterion (BIC) method.

### Resolution of fine-scale population structure

To reveal any fine-scale stratification between and among all populations, network analysis was carried out using the NetView P pipeline v.0.4.2.5 [64,65]. A population network was generated based on a shared allele 1- identity-by-similarity (IBS) distance matrix created in the PLINK v.1.07 toolset [66]. The network itself is constructed with the super-paramagnetic clustering (SPC) algorithm and Sorting Points Into Neighbourhoods (SPIN) software, which computes the maximum number of nearest neighbours for a given individual [64,65,67]. The network is then visualised and edited in the Cytoscape v.2.8.3 network construction package [68]. The IBS matrix and corresponding networks were constructed at various thresholds of the maximum number of nearest neighbour (k-NN) values between 5 and 40. Additionally, a hierarchical Analysis of Molecular Variance (AMOVA) was carried out in GenAlEx v.6.5 [69], to examine variation between farmed and wild groups of populations.

### Examination of adaptive variation

To detect signatures of selection, all pairwise population combinations were considered for *F*_st_ outlier detection. Testing failed to detect any outlier loci (see results), with the exception of three population pairs. Two independent outlier detection methods were used to identify candidate loci under selection, comprising the BayeScan v.2.1 [70,71] and LOSITAN selection detection workbench [72] packages. BayeScan 2.1 and LOSITAN employ different analytical approaches, and their joint use increased the statistical confidence of *F*_st_ outlier detection [16,73,74]. Jointly identified loci at high probability using both methods were considered to be statistically true outliers.

BayeScan 2.1 analyses were performed on a 1:10 prior odds probability for the neutral model and commenced with 20 pilot runs consisting of 5,000 iterations each. This was followed by 100,000 iterations with a burn-in length of 50,000 iterations [70]. Once probabilities had been calculated for each locus, the BayeScan 2.1 function *plot_R* was used in the *R* v.3.2.0 statistical package to identify putative outlier loci at various False Discovery Rates (FDR). A range of FDR values from 0.01 to 0.10 were evaluated based on preliminary testing, and recommendations by Ball [75] and Hayes [76]. All LOSITAN outlier detection was computed within a 95% confidence interval under an infinite allele model, with 50,000 iterations also evaluating a range of FDR values from 0.01 to 0.10 to match the BayeScan 2.1 analyses. All other test parameters remained at their default settings, with the exception of the 'Neutral' mean *F*_st_ and 'Force mean *F*_st_' options being enabled.

The results of the BayeScan 2.1 and LOSITAN analyses, together with the construction of pairs of Quantile-Quantile plots (QQ-plots) were used to assess the suitability of an FDR threshold for outlier detection between the two methods. The *R* package *GWASTools* v.1.14.0 [59,77] was used to construct all QQ-plots at all FDR levels examined. All loci were included in the first QQ plot constructed, to visualise deviation outside the bounds of a 95% confidence interval. If deviation was observed, a second plot was generated excluding all outlier loci. If all remaining loci were normally distributed, this was interpreted as confirmation that true outlier loci had been detected.

### Particle dispersal simulation

To independently compare results of the population genomic analyses with environmental data and to simulate larval transport pathways between sampling locations, a particle dispersal model was developed, which is publicly available at https://github.com/CyprienBosserelle/DisperGPU. Larvae typically remain in the plankton for 26–30 days prior to settlement [29,30], and due to very limited motility, are largely dispersed by current advection and turbulent diffusion in the ocean surface (mixed) layer.

### Hydrodynamic and dispersal numerical models

The particle dispersal model was driven by current velocity output from the global HYbrid Coordinate Ocean Model (HYCOM) data [78,79]. HYCOM is a global hydrodynamic model that simulates ocean surface heights, currents, salinity and temperature, both at the surface and at depth. The model is driven by meteorological forcing, and constantly constrained by the assimilation of global, remote and in-situ ocean observations. As the model simulates regional and global circulation, it does not include tidal or surface wind waves. HYCOM is highly useful for forecasting and simulation experiments, with public availability at https://hycom.org. The HYCOM model had a resolution of 1/12th of a degree and output every day. Although it simulates current movement in all three dimensions, only the surface layer was used to drive the dispersal model, as this is where larvae remain in the water column [80]. The particle model used a standard Lagrangian formulation [22,23], where particles have no physical representation, but rather track the displacement of neutrally buoyant small objects such as larvae (relative to the model resolution), at the ocean surface. Particle displacement is expressed as:

$$\Delta x=u_{p}*\Delta t+K$$ (1)

Here *x* represents particle position (latitude and longitude), Δ*x* is particle displacement during a time step Δ*t* (which was set at 1 hour), and *u*_*p*_ is the surface current speed at the location of the particle. *K* is the eddy diffusivity which takes account of the random displacement of the particle, due to turbulent eddies at a scale smaller than the hydrodynamics model resolution. *K* is calculated after Viikmäe et al. [81] as follows:

$$K=\sqrt{-4E_{h}\Delta t\;\log\left(1-R_{NA}\right)}\;\cos\left(2\pi R_{NB}\right)$$ (2)

Here *E*_*h*_ is a horizontal turbulent diffusion coefficient, and *R*_*NA*_ with *R*_*NB*_ are normally distributed random numbers. The horizontal turbulent diffusion coefficient is unknown, but assumed to be 1 m^2^s^-1^. *u*_*p*_ is calculated by interpolating the velocity from the hydrodynamics model, both spatially and temporally. Gridded surface currents are first interpolated to the dispersal step, after which the current velocity at each particle position is calculated using a bi-linear interpolation of the gridded surface currents, where only surface currents are taken into account and vertical movements neglected [82]. The particle age is retained and increases with simulation progression.

### Model configuration

Particles were seeded in eight locations broadly corresponding to locations from where oysters were sampled for genetic analyses (see Fig 4). Seeding locations were represented at scales larger than the sampling locations to factor in the extent of surrounding coral reef habitat and farm boundaries. All seed areas were also extended further offshore to account for the fact that the HYCOM model is not adapted for shallow water environments, and does not resolve fine-scale hydrodynamic patterns <10 km [83]. At each seed location, 25,600 particles were released once at the start of the simulation, which optimised the computational requirements for running the dispersal model.

The simulation was carried out using HYCOM data for February-April 2009 and 2010, based on observations of the peak spawning period for *P*. *margaritifera* in Fiji [84,85], and to test for circulation pattern differences over El Niño Southern Oscillation (ENSO) event years, (2009 recorded an El Niño). Selection of this timeframe was also based upon inference of when sampled oysters were likely to be completing larval development and undergoing settlement, using shell size to approximate age [86,87]. In this way, results of both the genetic and hydrodynamic analyses were restricted to the oysters sampled.

Particle positions were extracted at time intervals of 1, 15, 30 and 60 days post-seeding and no mortality or competency behaviour of the particles was simulated. Explicit, quantitative correlation of the genetic and hydrodynamic analyses was not possible, as this would have required genetic analysis of oysters at all potential source and sink locations with dense sampling coverage, and modelling of substantially more complex particle behaviour than computational resources permitted. Instead, an independent approach was adopted here, to examine congruency of results produced by the two analyses. Although the model is unsuitable for evaluation of recruitment rates, it does allow insights into possible connectivity between sampling locations.

## Results

### Genotyping and SNP discovery

Following sequencing, a total of 765,273,656 PE raw reads were obtained for all nine libraries across both lanes. Read filtering using the STACKs pipeline ('process_radtags' and 'ustacks' modules) to discard low quality reads (Phred33 score <30; 5.25% discarded), ambiguous barcodes and overrepresented sequences, resulted in 725,064,036 high quality reads remaining. These reads were used to generate a locus catalogue in the 'cstacks' module containing 303,650 stacks (S1 Table). This catalogue was used to generate all genotypes, using a median number of 555,524 reads to assemble 33,738 stacks for each individual (average read depth per stack of 17.81). Subsequent filtering at a minimum read depth of 10 per stack and MAF>0.02 resulted in a total of 42,341 genome-wide SNPs being genotyped. The primary dataset of 42,341 SNPs was screened to retain only the single most informative SNP per locus, remove those loci significantly deviating from HWE (p<0.00001) and under LD (p<0.0001) across all populations, retain individuals/populations with maximum genotyping rates, and also remove loci generated from contaminant sequences. These steps generated a final dataset of 4,123 high quality, polymorphic, genome-wide SNPs for further population genomic analyses.

### Population genomic diversity and differentiation

Observed heterozygosities were significantly lower (p<0.05) than expected heterozygosities for all populations (*H*_o_: 0.0621–0.1461; *H*_n.b._: 0.2903–0.3449, see Table 1**)**, and displayed similar trends to the proportions of rare alleles present in each population. The individual average multi-locus heterozygosity (MLH) computations matched the trends in observed heterozygosity, with the Kadavu (Ravitaki, wild) and Udu Point (wild) populations having the lowest (0.0687) and highest (0.1522) values, respectively. Lower MLH values were observed for island archipelago populations, when compared with oysters sampled from locations neighbouring larger land masses; e.g. Yasawa and the two Kadavu sites (0.0703, 0.0695 and 0.0687 respectively), vs. Ra, Raviravi and Udu Point (0.1407, 0.1465 and 0.1522, respectively). Similar patterns were apparent in the standardised heterozygosity (SH) metrics (Table 1), with island archipelago population SH values ranging from 0.5361–0.8899 (Kadavu; Galoa to Lau), and mainland populations producing values between 0.8249–1.1609 (Savusavu; Vatubukulaca to Udu Point).

**Table 1:** Genetic diversity indices for the wild and farmed *P*. *margaritifera* populations examined.

Population	Origin	n	Proportion of rare alleles (MAF <5%)	*N*_*eLD*_[95% C.I.]	*H*_*o*_(± SD)	*H*_n.b._(± SD)	*F*_is_(p<0.01)	MLH (± SD)	SH(± SD)	IR(± SD)
Ra (Namarai)	Farm (major island; Viti Levu)	50	11.3%	658.4[534.8–854.9]	0.1338(±0.1261)	0.2903(±0.1443)	0.4639	0.1407(± 0.0189)	1.1226(± 0.1623)	0.5105(± 0.0667)
Taveuni (Wailoa)	Farm (offshore island)	43	10.9%	∞[∞—∞]	0.1054(±0.1155)	0.2943 (±0.1507)	0.5513	0.1052(± 0.0699)	0.7383(± 0.3749)	0.6733(± 0.1780)
Raviravi	Farm (major island; Vanua Levu)	32	10.4%	∞[2422.5 - ∞]	0.1353(±0.1325)	0.2950 (±0.1488)	0.4552	0.1465(± 0.0221)	1.1414(± 0.1290)	0.4943(± 0.0813)
Savusavu (Vatubukulaca)	Farm (major island; Vanua Levu)	50	6.5%	∞[∞—∞]	0.0922(±0.1387)	0.3151 (±0.1414)	0.5239	0.1007(± 0.0469)	0.8249(± 0.4129)	0.6760(± 0.1511)
Savusavu (Wailevu)	Farm (major island; Vanua Levu)	49	8.6%	152.4[142.0–164.3]	0.1258(±0.1552)	0.3062 (±0.1430)	0.4903	0.1366(± 0.0149)	1.1138(± 0.1183)	0.5567(± 0.0537)
Savusavu (Wailevu, hatchery)	Farm (major island; Vanua Levu)	50	11.4%	5.2[5.1–5.3]	0.1380(±0.1860)	0.3063 (±0.1540)	0.4370	0.1456(± 0.0228)	1.1690(± 0.1727)	0.5713(± 0.0702)
Lau (Nayau Island)	Wild (archipelago)	50	9.8%	∞[∞—∞]	0.1093(±0.1176)	0.2975 (±0.1476)	0.5058	0.1111(± 0.0356)	0.8899(± 0.2815)	0.6189(± 0.1246)
Yasawa (Naviti Island)	Wild (archipelago)	35	7.0%	∞[∞—∞]	0.0653(±0.0956)	0.3113 (±0.1453)	0.6423	0.0703(± 0.0343)	0.5514(± 0.2783)	0.7613(± 0.1229)
Udu Point (Vunikodi)	Wild (major island; Vanua Levu)	18	7.4%	∞[∞—∞]	0.1461(±0.1535)	0.3169 (±0.1468)	0.4740	0.1522(± 0.0096)	1.1609(± 0.0708)	0.4972(± 0.0337)
Kadavu (Galoa Island)	Wild (archipelago)	25	3.8%	∞ [∞—∞]	0.0673(±0.1322)	0.3449 (±0.1380)	0.6407	0.0695(± 0.0311)	0.5361(± 0.2510)	0.7897(± 0.0950)
Kadavu (Ravitaki)	Wild (archipelago)	25	3.8%	∞ [∞—∞]	0.0621(±0.1131)	0.3444 (±0.1398)	0.6876	0.0687(± 0.0191)	0.5498(± 0.1564)	0.7907(± 0.0584)

The parameters calculated included proportion of rare alleles (<5%), effective population size by the linkage disequilibrium method (*N*_*eLD*_; 95% confidence intervals indicated within brackets), observed heterozygosity (*H*_o_), average expected heterozygosity corrected for population sample size(*H*_n.b._), inbreeding coefficient values (*F*_is_), average individual multi-locus heterozygosity (MLH), standardised heterozygosity (SH) and internal relatedness (IR).

Inbreeding coefficient (*F*_is_) values were variable across populations (Table 1), ranging from 0.4370 for the Savusavu hatchery population, to 0.6876 for the Kadavu (Ravitaki) wild population. Interestingly, the hatchery produced Savusavu oysters demonstrated the lowest *F*_is_ values, whereas several wild populations, such as Yasawa (0.6423) and Taveuni (0.5513), produced higher values. Generally, slightly higher *F*_is_ values were observed among populations sourced from island archipelagos, e.g. Taveuni, Yasawa and the two Kadavu sites (0.5513, 0.6423, 0.6407 and 0.6876, respectively). This contrasted with estimates for oysters collected from fringing reef systems connected with the major islands of Viti Levu and Vanua Levu; e.g. Raviravi, Ra, Udu Point and Wailevu at Savusavu (0.4552, 0.4639, 0.4740 and 0.4903, respectively). Internal relatedness (IR) was comparable to the *F*_is_ values calculated for each respective population. The highest IR values were observed for all island populations, ranging from 0.6189 (Lau) to 0.7907 (Kadavu, Ravitaki). Among the farmed populations, the Raviravi (0.4943), Ra (0.5105), Savusavu (Wailevu; 0.5567) and Savusavu (Wailevu hatchery; 0.5713) oysters exhibited intermediate IR values, while the highest IR was recorded for oysters sampled at Savusavu (Vatubukulaca; 0.6760).

Estimates of effective population sizes were infinite for all populations (Table 1), with the exception of the Ra (658.4; [95% CI: 534–854.9]), Savusavu (Wailevu; 152.4 [95% CI: 142–164.3]) and Savusavu hatchery oysters (5.2 [95% CI: 5.1–5.3]). Pearl oysters obtained from these locations were all farmed animals, and sourced from spat collector deployments adjacent to the farm sites. The only farm sites sampled which produced infinite *N*_*eLD*_ values were Taveuni and Ra, however, most of these animals had been directly collected from reef systems adjacent to the farms themselves. The Savusavu hatchery population was found to be bottlenecked with the lowest *N*_*eLD*_ of 5.2, most likely as a result of variable family survival and broodstock contributions.

Relatedness calculations between individuals revealed no parent-offspring pairs present in the dataset (S2 Table). However, full-sib and half-sib relationships were detected for the Savusavu (Vatubukulaca) farm population (with 8 full-sib and 86 half-sib pairs), and 83 full-sib and 116 half-sib pairs identified for the Savusavu hatchery-produced oysters. When between-region relationships were assessed by examining all populations together (S3 Table), the degree of relatedness declined with increasing geographic distance. The largest number of full-sib relationships was detected between Savusavu and Lau (25), with lower numbers between Savusavu and Kadavu, Taveuni and the Yasawa archipelago respectively, (four relationships each). Higher numbers of half-sib relationships between these regions were discovered, particularly between Savusavu and Lau, Taveuni, Kadavu, the Yasawa archipelago and Raviravi (73, 37, 24, 17 and 14 respectively). Between the most distant populations sampled, only 1–2 full-sib and 1–9 half-sib relationships were detected between the Yasawa and Lau, Taveuni and Kadavu populations, respectively. However, 19 half-sib relationships were evident between both Kadavu-Lau and Kadavu-Taveuni.

### Resolution of population structure

**Fig 2:**
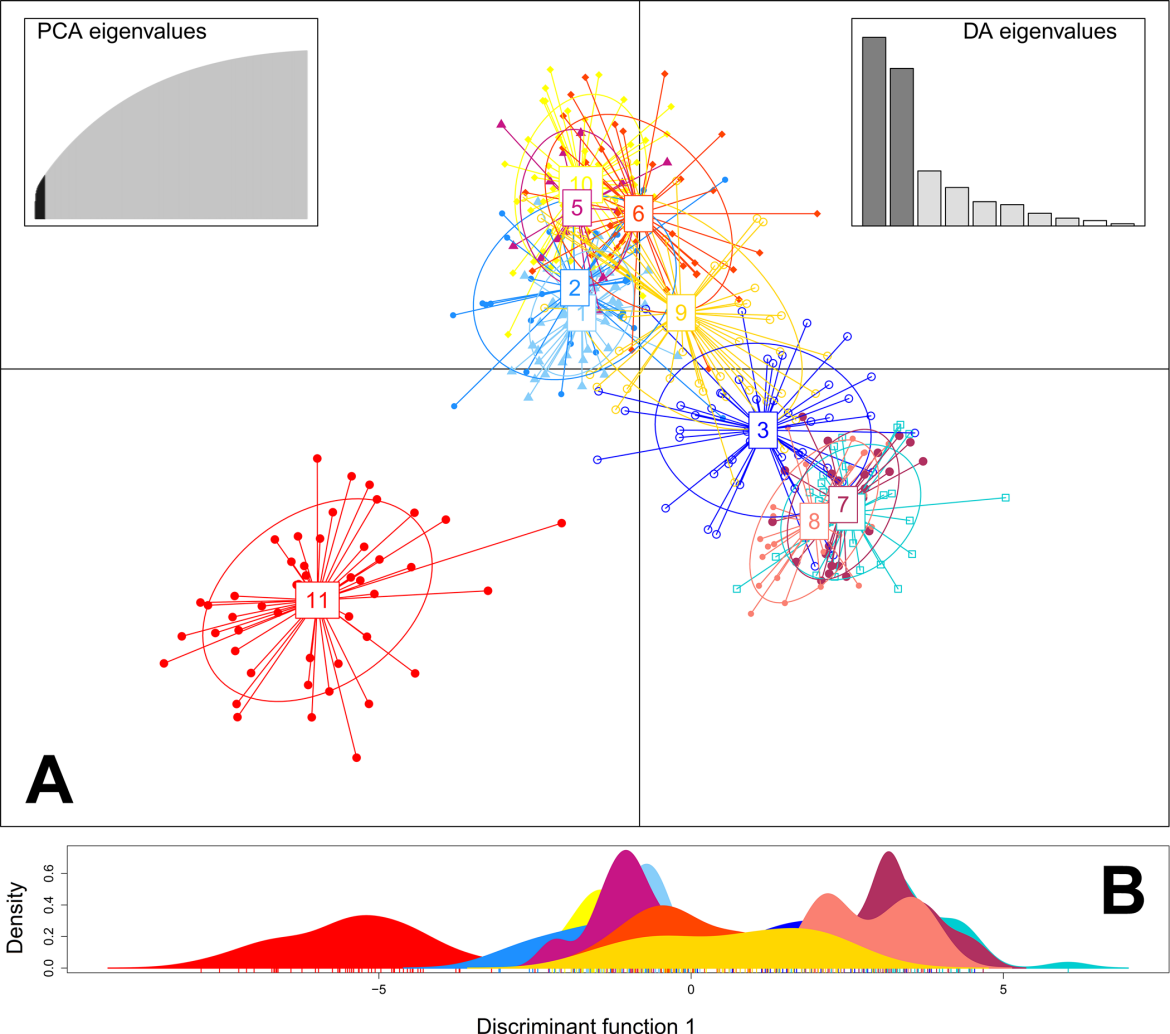
**Discriminant Analysis of Principal Components (DAPC) scatter plot (A) and individual density plot on the first discriminant function (B), drawn across 427 *P*. *margaritifera* individuals in the *R* package *adegenet***. Dots represent individuals, with colours denoting sampling origin and inclusion of 95% inertia ellipses. Site colours correspond with Fig 1, and site numbers are as follows: (1) farm site at Namarai, Ra; (2) farm site at Raviravi; (3) Lau group; (4) Yasawa group; (5) Udu Point; (6) Taveuni; (7) Kadavu (Galoa Island); (8) Kadavu (Ravitaki); (9) farm site at Savusavu (Vatubukulaca); (10) farm site at Savusavu (Wailevu) and (11) farm site at Savusavu (Wailevu, hatchery produced oysters).

Pairwise *F*_st_ estimates (S4 Table) did not significantly depart from zero across almost all populations (average overall *F*_st_ = 0.0028; p>0.05), except for the hatchery produced oysters (Savusavu, Wailevu), which showed weak, but significant separation (p<0.000001) from four other populations: Ra (farm), Raviravi (farm), Udu Point (wild) and Savusavu, Wailevu (farm). Evaluation of population structure with a DAPC following α-score optimisation to retain 16 informative principal components (S1 Fig), revealed differentiation across two separate clusters (Fig 2). The Savusavu hatchery oysters separated from all other populations, with all remaining populations forming a single, diffuse cluster with overlapping 95% inertia ellipses. This separation was confirmed by testing for the actual number of discrete clusters, which was determined to be k = 2 (Bayesian Information Criterion (BIC) method; S2 Fig).

**Fig 3:**
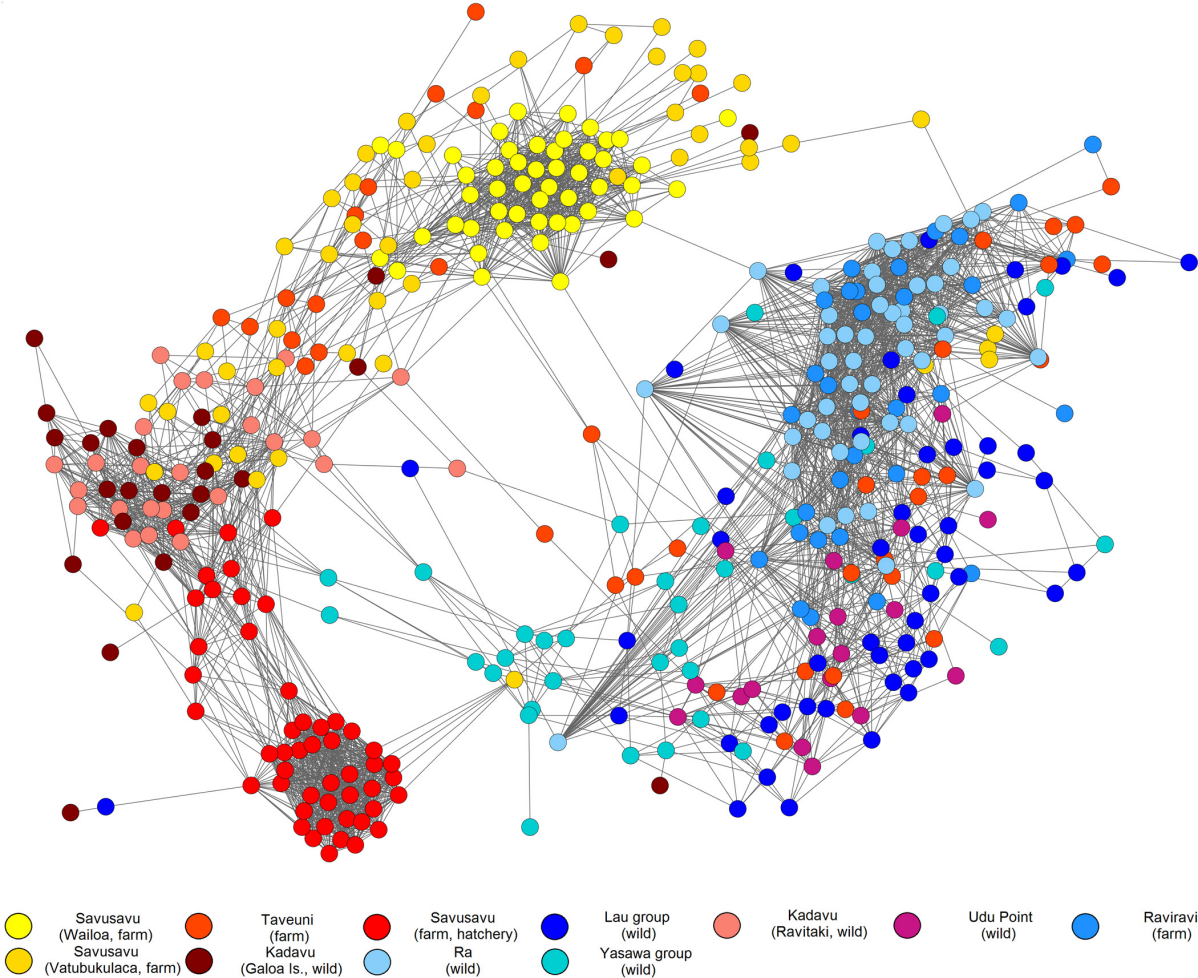
Population network of *P*. *margaritifera* individuals created using the Netview P v.0.4.2.5 pipeline after Steinig *et al*. [64]. The network has been visualised at a maximum number of nearest neighbour (k-NN) threshold of 40, using 4,123 SNPs and 427 individuals. Each dot represents a single individual, and population colours correspond with Figs 1 and 2.

Examination of fine-scale population sub-structure using the NetView P network (Fig 3) revealed a similar pattern of separation to the DAPC analysis, although with a greater level of individual resolution. Two large genetic groups were resolved, one of which incorporated six populations, while the other comprised a diffuse assemblage of the remaining five populations. The first group included the Savusavu (Wailevu) and Savusavu hatchery oysters, which formed two distinct clusters and remained separate from all other groups. Located between these two clusters, the two Kadavu, as well as the Taveuni and Savusavu (Vatubukulaca) populations also grouped together. The second larger group contained the Ra and Raviravi populations which formed a tight assemblage, along with a less compact cluster containing the Yasawa, Lau and Udu Point oysters. Connectivity between the two larger groups was limited to individuals belonging to the Yasawa, Taveuni, Savusavu (Vatubukulaca) and Lau populations. Identical trends were observed in networks constructed at lower k-NN values ranging from 5 to 35 (results not shown here), with the overall patterns of separation remaining consistent. Results of the hierarchical AMOVA were significant (p<0.001), and found that only 2% of the proportion of variation was attributable between wild and farm populations, whereas greater proportions were divided among individuals (68%), among populations (18%) and within individuals (12%).

### Examination of adaptive variation

Testing failed to detect any outlier loci, with the exception of three population pairs. Detection of *F*_st_ outlier loci at three FDR thresholds of 0.01, 0.05 and 0.10 for each of the pairwise population comparisons discovered between two and nine directional outlier SNPs jointly identified by Bayescan 2.1 and LOSITAN (Table 2). These pairwise population comparisons were carried out between Savusavu (Wailevu) and Lau, Udu Point and Kadavu (both populations considered together), as well as the Yasawa archipelago and Lau. These sites were located at maximum geographic distances across the Fiji Islands, positioned across environmental gradients (offshore island vs. mainland island and fringing vs. barrier reef habitats), as well as at opposing points along the major larval transport pathway identified from the particle dispersal simulation analysis. All directional outliers detected by Bayescan were also detected by LOSITAN, and no outlier loci were detected by either platform when all populations were considered together. Bayescan 2.1 analyses failed to detect any balancing outlier loci (zero or negative alpha values) for all pairwise population comparisons, and hence all balancing outliers reported were from LOSITAN computations. LOSITAN runs detected between 43 and 278 balancing loci across all three FDR thresholds for each pairwise population comparison. In order to select an FDR threshold for accepting a final number of outlier loci for each comparison, QQ plots were constructed for each dataset at all three thresholds. A final stringent FDR threshold of 0.01 was selected on the basis of the QQ plots (S3 Fig), under which 5, 3 and 2 directional outlier loci were detected between the Savusavu (Wailevu)-Lau, Udu Point-Kadavu and Yasawa-Lau pairwise population comparisons, respectively.

**Table 2:** Numbers of putative directional and balancing *F*_st_ outlier loci discovered. Tests were carried out at three False Discovery Rate (FDR) thresholds using BayeScan 2.1 [70] and LOSITAN [72]. Jointly-identified loci were identified using both outlier detection platforms.

		Directional			Balancing		
Populations compared	FDR	BayeScan 2.1	LOSITAN	Jointly-identified	BayeScan 2.1	LOSITAN	Jointly-identified
Savusavu, (Wailevu) and Lau	0.01	5	28	5	0	197	0
	0.05	8	46	8	0	206	0
	0.10	9	96	9	0	248	0
Udu Point and both Kadavu populations	0.01	3	21	3	0	43	0
	0.05	3	37	3	0	108	0
	0.10	4	56	4	0	84	0
Yasawa and Lau	0.01	2	18	2	0	201	0
	0.05	3	46	3	0	278	0
	0.10	4	61	4	0	241	0

To gauge the strength of the selection signal, the average *F*_st_ values for all directional and balancing outlier loci detected were examined at the selected FDR of 0.01. For the Savusavu (Wailevu)-Lau comparison, the average Bayescan 2.1 *F*_st_ value was 0.1168. Similarly, average *F*_st_ values of 0.1025 and 0.1496 were observed for the Yasawa-Lau, and Udu-Kadavu comparisons, respectively. The average LOSITAN *F*_st_ values for the balancing outliers detected remained consistent for the Savusavu (Wailevu)-Lau, Udu-Kadavu and Yasawa-Lau comparisons, (-0.0343, -0.0464 and -0.0426, respectively). Given this set of results, it appears that any signatures of selection if present, are too weak to be detected and/or indecipherable from the background signal. This was supported by contruction of neighbour joining trees to visualise population structure using directional outlier loci identified for each pairwise population comparison, based on 1-proportion of shared allele distances (results not shown here). All trees failed to show any separation between populations.

### Particle dispersal modelling

**Fig 4:**
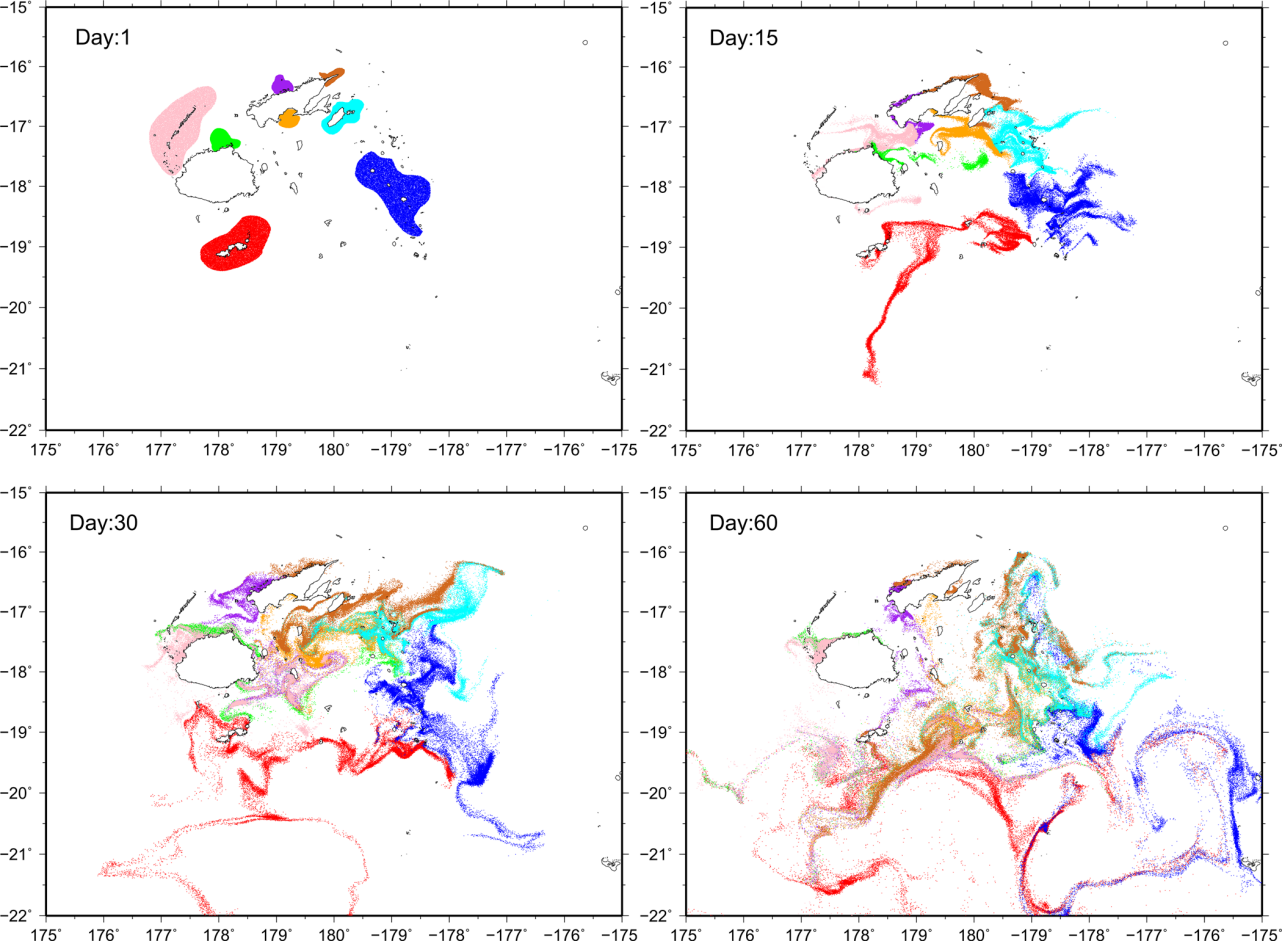
Results of 2009 particle dispersal simulation. Particle seed locations are shown in the day 1 position output, with the sampling regions colour coded as follows: Kadavu group (red), Yasawa group (pink), Ra (green), Raviravi (purple), Savusavu (orange), Udu Point (brown), Taveuni (light blue) and the central Lau group (dark blue). Simulated particle positions are shown at 15, 30 and 60 day outputs. An animation of dispersal simulation is provided as S4 Fig.

Simulation of larval transport pathways with the particle dispersal model demonstrated broad-scale mixture of larvae by surface ocean current systems operating within the Fiji Islands; (see Fig 4 for 2009 particle position outputs at 1, 15, 30 and 60 day time points and S4 Fig for an animation of the full dispersal simulation over 100 days. 2010 data were very similar to 2009 patterns and are not presented here). A singular dispersal corridor appears to initially drive larvae from all seed locations eastwards towards the Lau group of islands for a period of approximately 30 days; after which current movements oscillate across the centre of the Fiji group, while progressing in a southerly direction. Gene flow thus is likely to be homogenous between the Yasawa archipelago, Raviravi and Udu Point through the Bligh Water channel, towards sink locations in the Koro and Lau basins. Reef systems in the Lau group appear to receive recruits from all locations in Fiji, although varying degrees of self-recruitment are likely for the Udu Point, Raviravi and Yasawa populations, due to the prevailing current dynamics and architecture of the Great Sea Reef system north of Vanua Levu retaining larvae in those regions. Despite this, a portion of larvae originating in the Yasawa archipelago appear to recruit along the western coastline of Viti Levu and Ra. Similarly, larvae which are exported from Udu Point and Raviravi may mix with individuals from Savusavu and Taveuni. The lowest degree of mixing is likely to occur between populations located along a North-South axis (e.g. Udu Point and Kadavu), as the dominant dispersal pathway operates in a West to East direction. Interestingly, the simulation indicates that if larvae advected from Kadavu and Lau survive beyond 40 days post-hatching, it may be possible for a few individuals to recruit eastwards onto the reefs of Tongatapu in the Kingdom of Tonga, (approximate position -175° longitude; see Day 60 output in Fig 4).

### Discussion

By independently evaluating population genomic analyses with hydrodynamic dispersal simulation, we identified that Fijian *P*. *margaritifera* display a very shallow pattern of population structure, and are highly likely to constitute a single, biologically significant stock for fishery management. While diffuse patterns of population differentiation are apparent given the resolution of 4,123 SNPs used, the overall pairwise *F*_st_ estimates are small and not statistically significant (average overall *F*_st_ = 0.0028; p>0.05). Given the largely homogenising larval mass transport pattern resolved using hydrodynamic dispersal simulation and the levels of relatedness between populations, the pattern of structure detected plausibly reflects fine-scale differentiation at the generational and family levels, together with small, isolated patches of localised recruitment [32]. Furthermore, examination of loci under selection failed to detect any signatures of local adaptation, suggesting that environmental differences among populations are insufficiently heterogeneous to drive selection at the spatial scale examined (<400 km). Additionally, if weak local adaptation is present, the very high levels of gene flow between populations would likely override discernible signatures of selection. These results demonstrate the utility of independent population genomic and biophysical datasets for providing insights into the biology and ecology of a broadcast spawning bivalve, and have great potential for application to other marine species with similar life histories, where patterns of genetic structure and connectivity may not be well understood.

### Resolution of population structure, diversity and relatedness

A weak pattern of population structure with high levels of connectivity was evident among all populations sampled using both broad-scale (DAPC) and fine-scale (NetView P) methods, mirroring the results of a previous study in Fiji [17]. Investigations of *P*. *margaritifera* populations elsewhere have yielded similar results, including French Polynesia [31,32] and Japan [88]. Considering that *P*. *margaritifera* is a broadcast spawner with a relatively long PLD of between 26–30 days [29,30], the degree of larval mixing driven by surface ocean currents (as demonstrated by the hydrodynamic dispersal simulation), supports the finding that Fijian oysters from all 11 locations sampled may be classified as a singular genetic entity.

Population pairwise *F*_st_ estimates indicated shallow and non-significant levels of structure, with the hatchery-produced oysters being the only population demonstrating detectable differentiation. This is not surprising considering that this population had undergone a genetic bottleneck through limited broodstock use, and differential larval mortality typical of hatchery rearing conditions. DAPC with BIC analysis, and NetView P network analysis both resolved similar cluster patterns, and overall patterns correlated well with *F*_st_ results and larval transport pathways inferred from particle dispersal simulation.

The levels of observed heterozygosity (*H*_o_) detected were lower than expected across all populations (Table 1), keeping with the trend of heterozygote deficiency previously observed for *P*. *margaritifera* in Fiji [17], French Polynesia [31–34,89] and Japan [88]. Heterozygote deficits appear to be characteristic of a number of marine molluscs [90–92], and in the current study are also likely due to a technical artefact associated with RADseq-based genotyping approaches, where restriction enzyme cut site polymorphisms may cause allelic dropouts [56,57]. While stringent filtering measures were used to reduce the proportion of null alleles present in the final dataset, thorough testing of their effect on *H*_o_, *F*_is_, *N*_*eLD*_ and population differentiation estimates following the methods of Lal et al. [17] for *P*. *margaritifera*, revealed no impact on these metrics.

When assessing populations separately, estimates of individual average multi-locus heterozygosity (MLH), standardised heterozygosity (SH), inbreeding coefficient (*F*_is_) and internal relatedness (IR) agreed with trends observed in *H*_o_, which generally showed a lower diversity among pearl oysters sampled from island archipelago populations, compared to those from the larger land masses of Viti Levu and Vanua Levu (e.g. Av.MLH for the Kadavu (Galoa Island) and Raviravi (Vanua Levu) populations were 0.0695 cf. 0.1465 respectively). This observation may indicate higher rates of self-recruitment among island archipelago populations, and fits a growing body of evidence supporting significant self-recruitment for a number of broadcast spawning coral and reef fish species, with geographic setting strongly influencing the degree of larval retention within populations [93].

Patterns detected in the NetView P network, relatedness analyses and dispersal simulation indicate support for this observation, as geographically distant populations clustered separately (e.g. Kadavu and Taveuni island sites), and shared fewer pairwise family relationships than others with higher degrees of connectivity either through proximity (e.g. Ra and Raviravi), or position within the major current pathway (e.g. Yasawa and Lau). This was particularly evident between populations <150 Km apart containing 17–73 half-sibs, whereas populations situated farther apart held only 1–9. Examination of pairwise relationships between individuals within populations identified a larger number of full-sib and half-sib relationships for the bottlenecked hatchery produced population, as well as one farmed population sourced from spat collectors. For the latter, it is feasible that several individuals from one or more families remained poorly mixed in the plankton, and subsequently settled together on the spat collectors. This was suggested by Knutsen et al. [94] for their study on Atlantic cod, and similar variability has been observed in hatchery-produced *P*. *maxima* [90,95].

Assessments of *N*_*eLD*_ and individual pairwise relationships within populations indicated a generally high degree of connectivity between populations. However, reduced *N*_*eLD*_ was detected for three farmed populations, one of which was a hatchery-produced cohort that had experienced a genetic bottleneck as a result of standard hatchery spawning practices [17,88,90,95]. A possible explanation for the lower *N*_*eLD*_ observed for the two other populations may be differential settlement and survival on the spat collectors these oysters were collected from, as previous studies have shown highly variable settlement, survival and predation rates of newly settled *P*. *margaritifera* spat on collector gear [96–99].

The use of hydrodynamic modelling in parallel with genome-wide data for farmed and wild populations, adds fresh perspective for understanding the interaction of geographic and oceanographic influences contributing to population genetic structure in *P*. *margaritifera*. Studies on the genetic stock structure of this species predominantly originate in French Polynesia, where oysters are found in three distinct types of reef environments [31,34]. These comprise high island lagoons with fringing and barrier reef systems with open oceanic circulation (similar to those found in Fiji), atoll lagoons also with open circulation, and closed atoll lagoons with highly reduced circulation [31,32,34]. Lemer and Planes [31] detected connectivity at both small (less than 500 km) and large (greater than 1500 km) spatial scales between French Polynesian archipelagos which had open oceanic circulation patterns, mirroring the results of our observations for Fijian populations, but also found significant genetic structure for oysters contained within closed atoll lagoons.

### Examination of adaptive variation

Understanding levels of adaptive variation is critical for management of translocation, population supplementation and/or assisted migration, in order to avoid negative consequences such as outbreeding depression that may result from moving individuals into an environment they may be maladapted to [100,101]. This latter consideration is especially important for aquaculture, as productivity is heavily reliant on stock fitness [102–104]. Knutsen et al. [94] in their study on Atlantic cod also failed to detect signatures of selection, despite the species having an extensive North Atlantic natural distribution over known salinity and temperature clines. An explanation they offer for this finding is that their work examined a restricted geographical range, where environmental differences may be small, relative to conspecifics occupying more heterogeneous habitats over the broader species distribution. The situation may be similar for *P*. *margaritifera* in the present study, and examination of populations across larger spatial scales beyond the Fiji Islands should provide further insights.

The inability of *F*_st_ outlier testing to discern signatures of selection possibly indicates that the environments oysters were sampled from may be insufficiently heterogeneous to drive local adaptation at an easily detectable threshold. Further considerations include the type of trait under selection (polygenic or monogenic), as well as the opposing dynamics of gene flow against the strength of selection. That is, where local adaptation is present, it may be too weak to be detected by the SNP marker set used and lost to background noise. Nayfa and Zenger [11] examined three populations of the closely related silver-lip pearl oyster *P*. *maxima*, from Bali, West Papua and Aru in Indonesia, which were subject to a complex system of prevailing and seasonally reversing surface ocean currents. Evidence of directional selection was detected despite high levels of gene flow, causing divergence between oysters from Bali and West Papua against those from Aru, and the recommendation for aquaculture was to manage the Aru population separately from Bali and West Papua.

### Particle dispersal modelling

Examination of larval dispersal patterns using hydrodynamic modelling alone has been used for a number of marine taxa [105,106], including *P*. *margaritifera* [107], but comparatively few studies have sought to combine larval dispersal data with genome-wide population information. Among studies which have coupled oceanographic and genetic methods are White et al. [108], Galindo et al. [21] and Dao et al. [24] using microsatellite loci, however, the limited number of these markers have provided finite information about fine-scale population structure and adaptive variation [109,110].

The discovery of homogenised surface ocean current movement towards the Lau archipelago is well supported by the results of population genomic analyses presented here, particularly regarding broad and fine-scale population differentiation, genetic diversity levels and lack of adaptive variation within and among populations. It is interesting that the major larval sink location is situated in the Lau archipelago, which retains consistency across ENSO years. Further examination of fine-scale larval transport pathways is warranted to determine the degree of mixing within the Lau group, and to see if any settlement heterogeneity occurs there. Unfortunately, this was beyond the capability of the HYCOM hydrodynamic model used here, as the data is not captured at a resolution finer than a grid size of 10 km^2^ [79,83]. The HYCOM model is the only hydrodynamic model available for the Fiji Islands, however, given the future availability of a finer resolution model, gaining these insights is possible.

For broadcast spawning marine taxa with extended PLD, the inclusion of hydrodynamic dispersal data to better understand population connectivity in the marine environment is indispensable, as assessment of the magnitude of larval movements, along with patterns of current-driven differential recruitment may become possible. Work by Thomas et al. [107] in French Polynesia on connectivity between populations discovered that larval sink and source locations for *P*. *margaritifera* accounted for 26% and 59% of the variation observed respectively, underscoring its importance for larval supply and management of farmed and wild pearl oysters.

### Implications for fishery management

The persistent problem in stock assessment investigations of determining "biologically meaningful" genetic divergence between populations requires careful evaluation on a case by case basis, with respect to the biological questions being answered [3], fishery management goals and the characteristics of the organism(s) involved [4,94]. For high gene flow species where fine-resolution population genomic analyses detect weak divergence by examining neutral and adaptive variation, the use of independent environmental data provides important additional knowledge for informed fishery management decision making.

Given the findings of non-significant population differentiation and the absence of signatures of selection or apparent phenotypic differences among populations, these data support the existence of a singular, biological stock in the Fiji Islands. This suggests that fishery management of *P*. *margaritifera* in Fiji may be based upon treatment of all populations sampled here as one cohesive unit. Further evidence of this is found in the independent assessment of population connectivity by hydrodynamic dispersal simulation, which confirms broad scale panmixia across all populations. This finding is promising for developing aquaculture of this species in the country, as it may mean that spat collected in locations which freely exchange recruits can also be grown-out among them (e.g. Kadavu, Ra, Savusavu, Taveuni and Lau). For those populations which experience less connectivity (e.g. Yasawa, Raviravi and Udu Point), further investigation is required to determine if any negative consequences may result from either keeping these groups isolated, or opening them up to translocation.

The small spatial scale of the Fiji Islands and high levels of gene flow apparent for Fijian *P*. *margaritifera*, may actually facilitate uncomplicated fishery management and aquaculture development of this species in the country, compared to other locations such as French Polynesia, where oysters are distributed over larger scales and across heterogeneous habitats [31]. For French Polynesian populations, Lemer and Planes [34] and Arnaud-Haond et al. [33] reported that farmed populations originally sourced from genetically distinct wild oysters over a period of 20 years, had accumulated higher levels of genetic diversity than their progenitors, potentially providing a risk of outbreeding depression for wild oysters interbreeding with farmed individuals. While it is unlikely that a similar situation could occur for Fijian *P*. *margaritifera*, there are important lessons to be learnt from the French Polynesian experience. If hatchery production of spat outpaces the collection of wild spat as the primary source of oysters for grow out in the future, any potentially negative consequences as a result of genetic pollution effects could be minimised by careful selection of broodstock to maintain levels of genetic fitness.

## Conclusions

The use of genome-wide SNP data and hydrodynamic particle dispersal modelling have provided valuable insights into the population structure and connectivity of the black-lip pearl oyster in the Fiji Islands, filling a substantial knowledge gap on the stock structure of this species in the country. Simulation of larval transport with hydrodynamic dispersal modelling confirmed the existence of broad-scale connectivity by surface ocean current systems, correlating very well with patterns of differentiation, heterozygosity and adaptive variation discovered in the genetic data. There is strong support for the existence of a singular stock structure in the Fiji Islands, which is promising for developing aquaculture of this species in the country, as it indicates that germplasm transfer is possible between locations which freely exchange recruits. The combined use of both selectively neutral and loci under selection to elucidate fine-scale population variability (or the lack thereof), has high utility for stock assessment in high gene flow species, where biologically meaningful levels of divergence are not immediately apparent. Furthermore, independent assessment of connectivity using environmental data such as particle dispersal simulation, can provide valuable additional information for making fishery management decisions, when patterns in genetic data don't easily lend themselves to the identification of stock boundaries. Our study highlights the value of using both genomic and hydrodynamic data, for a comprehensive understanding of population structure and connectivity in broadcast-spawning marine taxa, and utilising the information collectively for aquaculture and sustainable fishery management.

## Supporting Information

S1 Fig α-score optimisation graph for generation of the Discriminant Analysis of Principal Components (DAPC) scatter plot. An optimal number of 16 principal components were suggested for retention using this analysis, based on 4,123 SNP loci in the *R* package *adegenet* [59,62,63].
(PDF)

S2 Fig Determination of the number of clusters following generation of the DAPC scatter plot using 4,123 SNP loci. An optimal number of k = 2 was suggested based on the BIC method implemented in the find.clusters function of the *R* package *adegenet* [59,61,62].
(PDF)

S3 Fig Verification of outlier loci detected for population pairwise comparisons using Quantile-Quantile plots (QQ plots) at an FDR of 0.01. Comparisons shown are for Savusavu-Lau (left), Udu Point-Kadavu (middle) and Yasawa-Lau (right). QQ plots are arranged in pairs with the top row displaying the p value distributions of all SNP loci while the bottom row displays the distribution when all outlier loci are removed. The red line indicates y = x linearity for conformity to a normal distribution, with the surrounding grey area approximating a 95% confidence interval.
(TIF)

S4 Fig Animation of full particle dispersal model simulation run for 2009 over 100 days. Particle seed location colour codes are identical to those described in **Fig 4**. [See.GIF file. Please note that the.GIF file needs to be opened in a web browser to display correctly.]
(GIF)

S1 Table Sequencing recovery rates and SNP identification at each filtering step in the STACKs 1.20 pipeline. (DOCX)

S2 Table Estimates of relationships. Values are provided for relationships between individuals within eleven Fijian populations of *P*. *margaritifera*, with 4,123 SNP loci using ML-RELATE [55]
(DOCX)

S3 Table Estimates of full-sib, half-sib and parent-offspring relationships. Estimates are provided between geographic regions sampled from eleven Fijian populations of *P*. *margaritifera* with 4,123 SNP loci using ML-RELATE [55]. All other between-region relationships examined indicated that individuals were unrelated.
(DOCX)

S4 Table Population pairwise *F*_st_ estimates. Estimates were computed using Arlequin [48] (Weir and Cockerham 1984 unbiased method), for 4,123 SNP loci in *P*. *margaritifera* from 11 Fijian populations. Significantly different values at p<0.000001 following 10,000 permutations are indicated with an asterisk.
(DOCX)
