# Supplementary figures and images for: A Parallel Population Genomic and Hydrodynamic Approach to Fishery Management of Highly-Dispersive Marine Invertebrates: The Case of the Fijian Black-Lip Pearl Oyster Pinctada margaritifera
Source: PLoS One. 2016 Aug 25;11(8):e0161390. doi: 10.1371/journal.pone.0161390 (PMC4999145; doi:10.1371/journal.pone.0161390)

# a-score optimisation – spline interpolation

Optimal number of PCs: 16

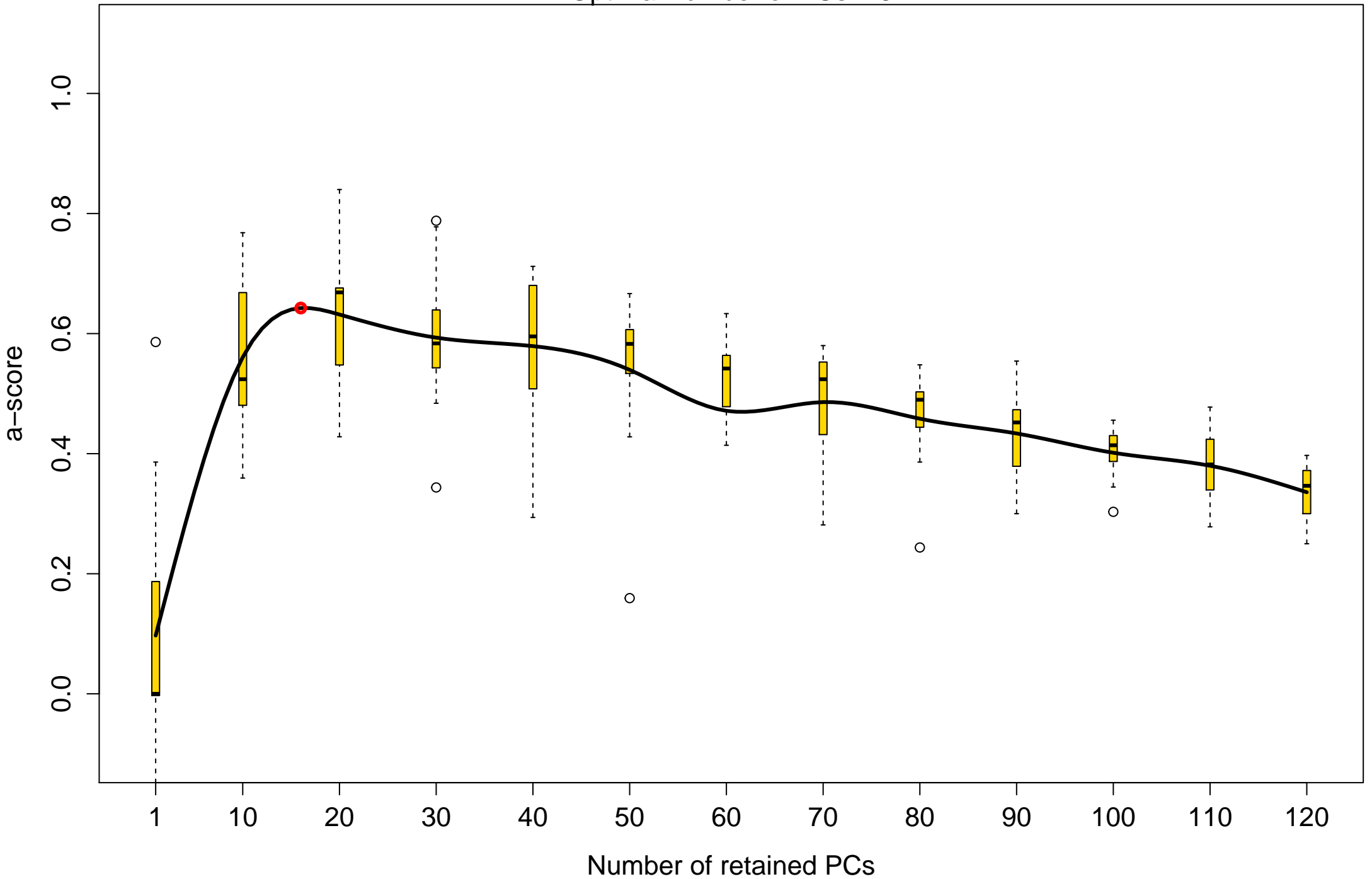

Supplement: S1 Fig — An optimal number of 16 principal components were suggested for retention using this analysis, based on 4,123 SNP loci in the R package adegenet [59,62,63]. (PDF) [file pone.0161390.s001.pdf]

# Number of cluster detection based on BIC

'X' indicates the actual number of clusters

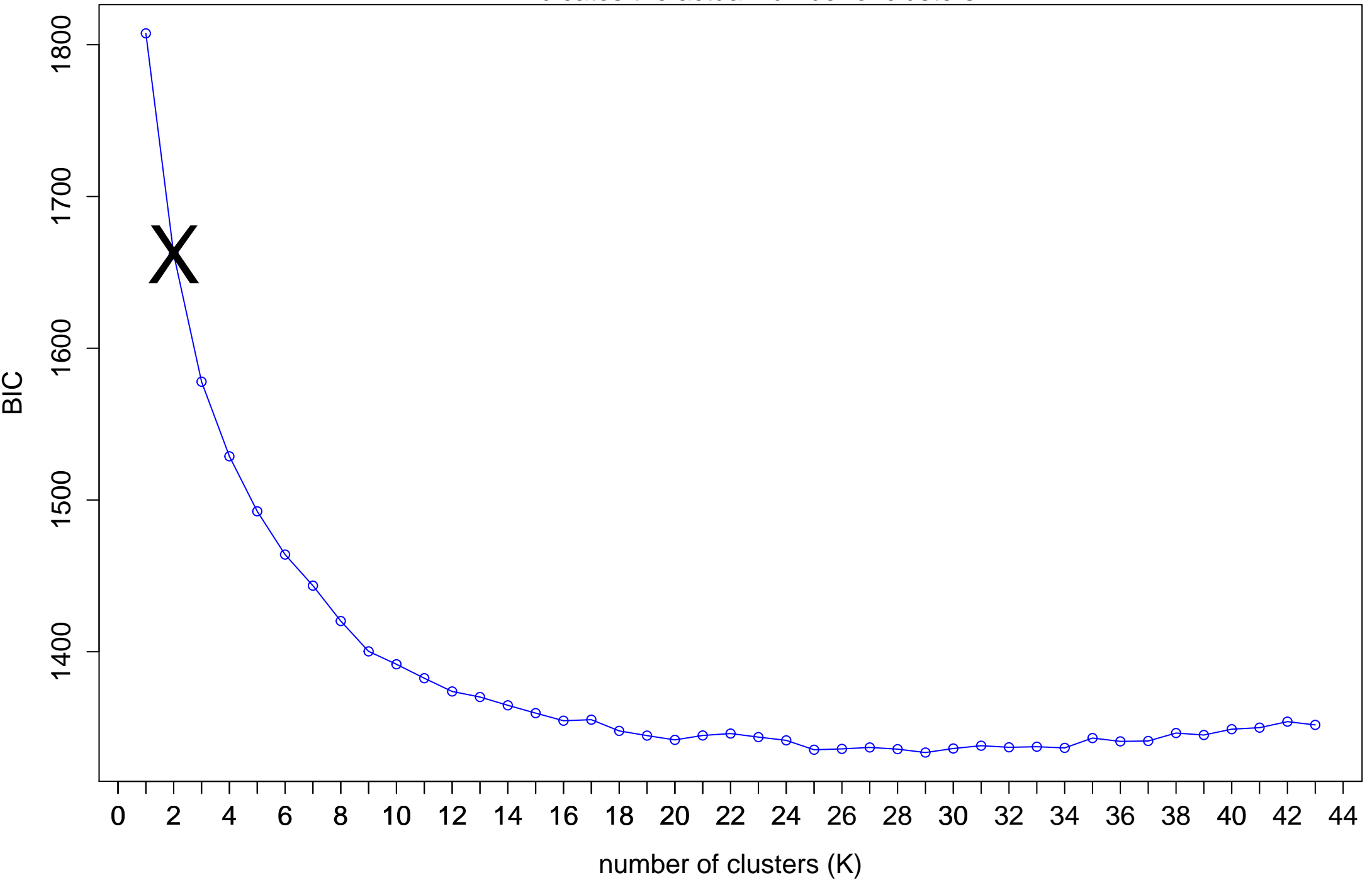

Supplement: S2 Fig — An optimal number of k = 2 was suggested based on the BIC method implemented in the find.clusters function of the R package adegenet [59,61,62]. (PDF) [file pone.0161390.s002.pdf]

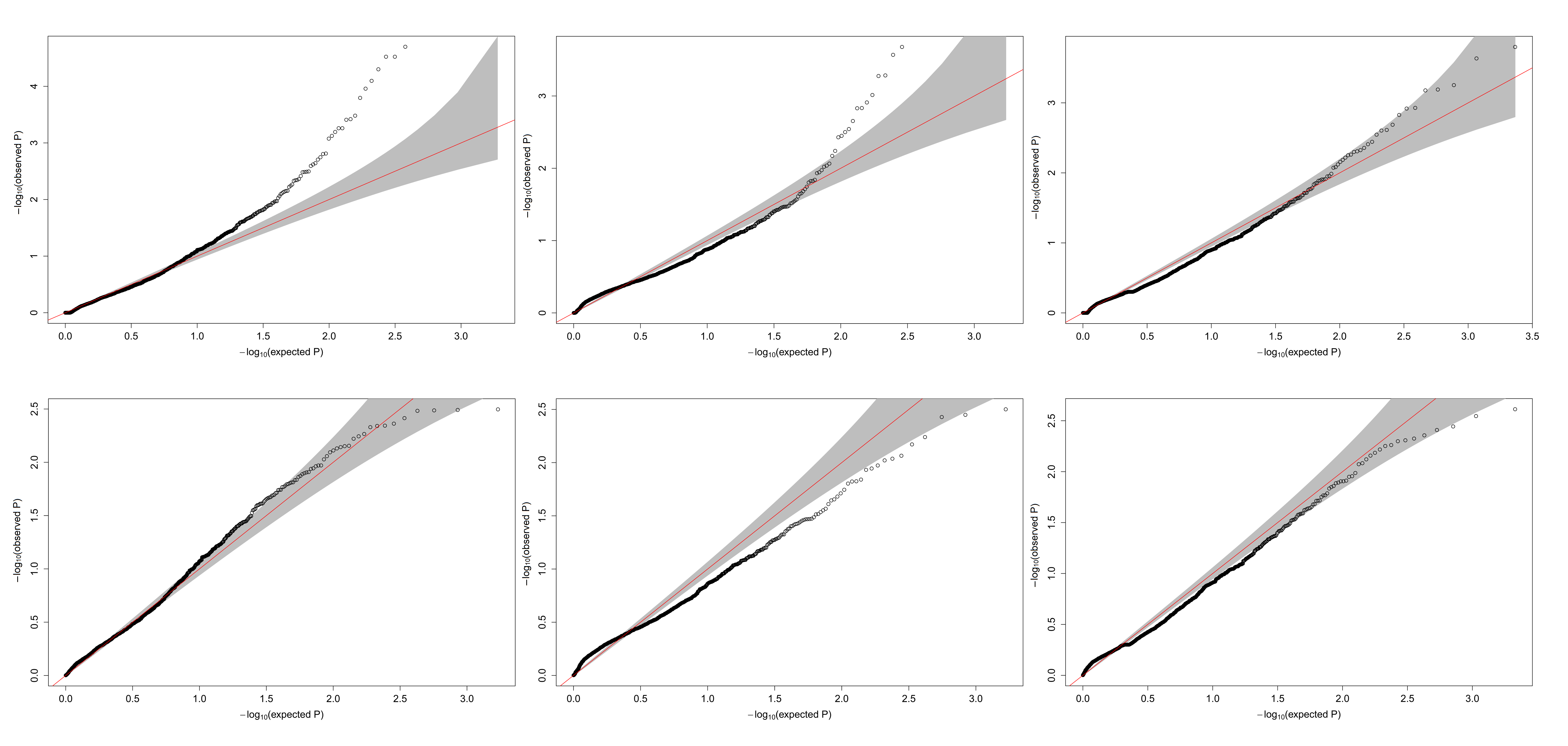

Supplement: S3 Fig — Comparisons shown are for Savusavu-Lau (left), Udu Point-Kadavu (middle) and Yasawa-Lau (right). QQ plots are arranged in pairs with the top row displaying the p value distributions of all SNP loci while the bottom row displays the distribution when all outlier loci are removed. The red line indicates y = x linearity for conformity to a normal distribution, with the surrounding grey area approximating a 95% confidence interval. (TIF) [file pone.0161390.s003.tif]

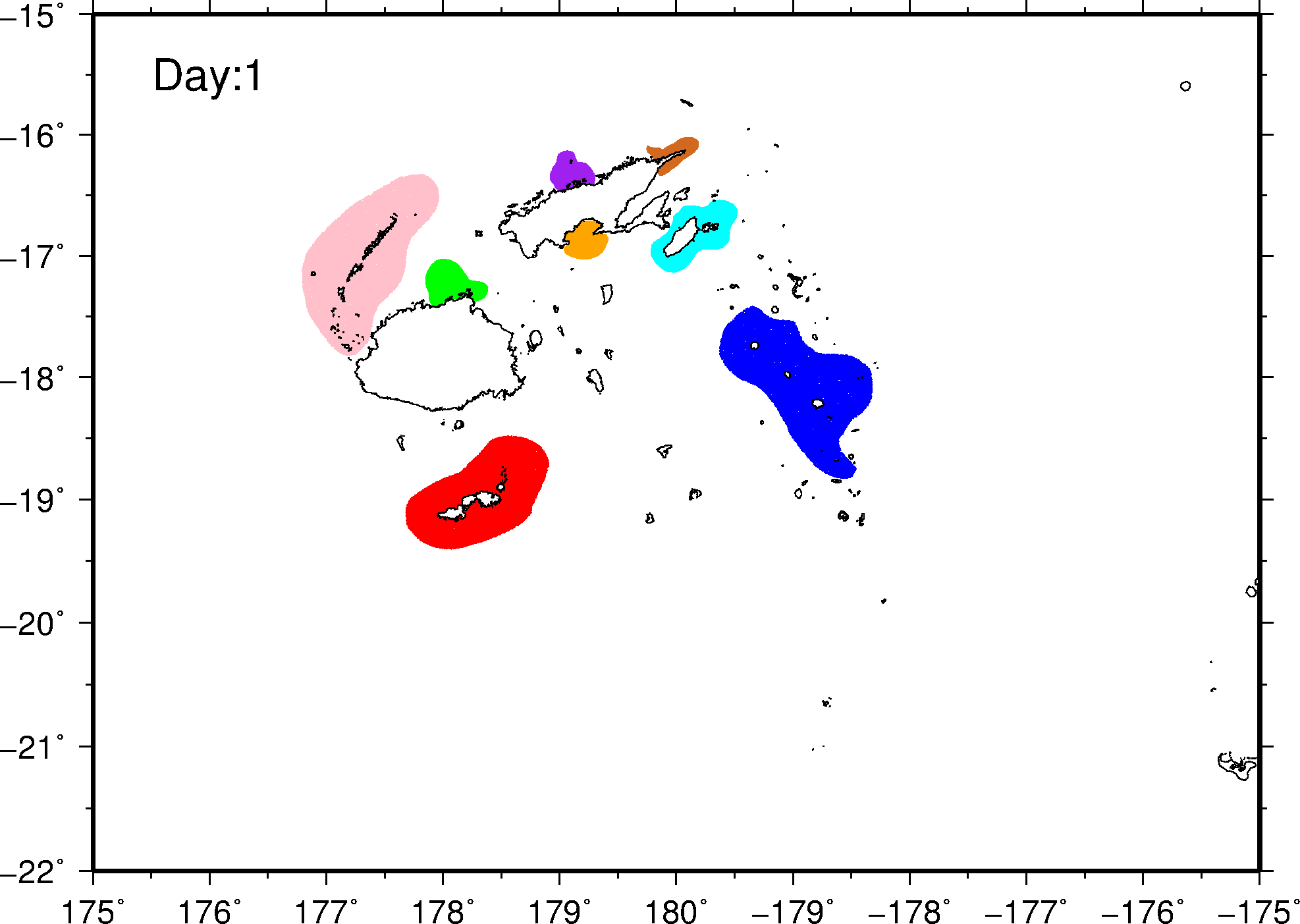

Supplement: S4 Fig — Particle seed location colour codes are identical to those described in Fig 4. [See.GIF file. Please note that the.GIF file needs to be opened in a web browser to display correctly.] (GIF) [file pone.0161390.s004.gif]
