# Supplementary material for: A Parallel Population Genomic and Hydrodynamic Approach to Fishery Management of Highly-Dispersive Marine Invertebrates: The Case of the Fijian Black-Lip Pearl Oyster Pinctada margaritifera
Source: PLoS One. 2016 Aug 25;11(8):e0161390. doi: 10.1371/journal.pone.0161390 (PMC4999145; doi:10.1371/journal.pone.0161390)
Supplement: S1 Table — (DOCX) [file pone.0161390.s005.docx]

**S1 Table**. **Sequencing recovery rates and SNP identification at each filtering step in the STACKs 1.20 pipeline.**

| **Metric** | **Number** |
| --- | --- |
| Number of raw reads | 765,273,656 |
| Number of retained reads | 725,064,036 |
| Overall number of stacks | 303650 |
| Number of SNP loci retained | 42,341 |
| Average number of stacks per individual | 33,738.94 |
| Average read depth per stack | 17.81 |
| Range of numbers of stacks | 2,062 - 95,560 |
| Range of stack depths | 8.47 - 51.94 |
