# Supplementary material for: A Parallel Population Genomic and Hydrodynamic Approach to Fishery Management of Highly-Dispersive Marine Invertebrates: The Case of the Fijian Black-Lip Pearl Oyster Pinctada margaritifera
Source: PLoS One. 2016 Aug 25;11(8):e0161390. doi: 10.1371/journal.pone.0161390 (PMC4999145; doi:10.1371/journal.pone.0161390)
Supplement: S2 Table — Values are provided for relationships between individuals within eleven Fijian populations of P. margaritifera, with 4,123 SNP loci using ML-RELATE [55] (DOCX) [file pone.0161390.s006.docx]

**S2 Table**. **Estimates of relationships between individuals** **using ML-RELATE [55].**

| **Population** | **Total # relationships**  **tested** | **Unrelated relationships** | **Full sib relationships** | **Half sib relationships** | **Parent-offspring relationships** |
| --- | --- | --- | --- | --- | --- |
| Ra  (Farm) | 1225 | 1223 | 1 | 1 | 0 |
| Raviravi  (Farm) | 496 | 496 | 0 | 0 | 0 |
| Lau  (Wild) | 1225 | 1220 | 0 | 5 | 0 |
| Yasawa  (Wild) | 595 | 593 | 2 | 0 | 0 |
| Udu Point  (Wild) | 153 | 153 | 0 | 0 | 0 |
| Taveuni  (Farm) | 903 | 902 | 0 | 1 | 0 |
| Kadavu, Galoa  (Wild) | 300 | 300 | 0 | 0 | 0 |
| Kadavu, Ravitaki  (Wild) | 300 | 299 | 0 | 1 | 0 |
| Savusavu, Vatubukulaca  (Farm) | 1225 | 1131 | 8 | 86 | 0 |
| Savusavu, Wailevu  (Farm) | 1176 | 1174 | 1 | 1 | 0 |
| Savusavu, Wailevu  (Farm; hatchery) | 1225 | 1026 | 83 | 116 | 0 |
