# Supplementary material for: A Parallel Population Genomic and Hydrodynamic Approach to Fishery Management of Highly-Dispersive Marine Invertebrates: The Case of the Fijian Black-Lip Pearl Oyster Pinctada margaritifera
Source: PLoS One. 2016 Aug 25;11(8):e0161390. doi: 10.1371/journal.pone.0161390 (PMC4999145; doi:10.1371/journal.pone.0161390)
Supplement: S3 Table — Estimates are provided between geographic regions sampled from eleven Fijian populations of P. margaritifera with 4,123 SNP loci using ML-RELATE [55]. All other between-region relationships examined indicated that individuals were unrelated. (DOCX) [file pone.0161390.s007.docx]

**S3 Table**. **Estimates of full-sib, half-sib and parent-offspring relationships between geographic regions using ML-RELATE [55].**

|  | Yasawa-Lau | Yasawa-Raviravi | Yasawa-Taveuni | Yasawa-Udu Point | Kadavu-  Udu Point | Kadavu-Taveuni | Kadavu-Lau | Kadavu-Yasawa | Kadavu-Raviravi | Kadavu-Ra | Taveuni-Ra | Taveuni-Raviravi |
| --- | --- | --- | --- | --- | --- | --- | --- | --- | --- | --- | --- | --- |
| Full sib relationships | 1 | 0 | 1 | 0 | 0 | 2 | 2 | 2 | 0 | 0 | 0 | 0 |
| Half sib relationships | 1 | 4 | 7 | 1 | 2 | 19 | 19 | 9 | 6 | 2 | 1 | 2 |
| Parent-offspring relationships | 0 | 0 | 0 | 0 | 0 | 0 | 0 | 0 | 0 | 0 | 0 | 0 |
|  | Savusavu-Ra | Savusavu-Udu Point | Savusavu-Kadavu | Savusavu-Raviravi | Savusavu-Lau | Savusavu-Taveuni | Savusavu-Yasawa | Lau-  Ra | Lau-Raviravi | Lau-Taveuni | Lau-Udu Point |  |
| Full sib relationships | 0 | 0 | 4 | 0 | 25 | 4 | 4 | 0 | 0 | 0 | 0 |  |
| Half sib relationships | 5 | 6 | 24 | 14 | 73 | 37 | 17 | 3 | 4 | 5 | 1 |  |
| Parent-offspring relationships | 0 | 0 | 0 | 1 | 0 | 0 | 0 | 0 | 0 | 0 | 0 |  |
