# Supplementary material for: A Parallel Population Genomic and Hydrodynamic Approach to Fishery Management of Highly-Dispersive Marine Invertebrates: The Case of the Fijian Black-Lip Pearl Oyster Pinctada margaritifera
Source: PLoS One. 2016 Aug 25;11(8):e0161390. doi: 10.1371/journal.pone.0161390 (PMC4999145; doi:10.1371/journal.pone.0161390)
Supplement: S4 Table — Estimates were computed using Arlequin [48] (Weir and Cockerham 1984 unbiased method), for 4,123 SNP loci in P. margaritifera from 11 Fijian populations. Significantly different values at p<0.000001 following 10,000 permutations are indicated with an asterisk. (DOCX) [file pone.0161390.s008.docx]

**S4 Table.** **Population pairwise *F*_st_ estimates (Weir and Cockerham 1984 unbiased method), computed using Arlequin 3.5.1.3 [48].**

|  | Ra  (Farm) | Raviravi  (Farm) | Lau  (Wild) | Yasawa  (Wild) | Udu Point  (Wild) | Taveuni  (Farm) | Kadavu, Galoa  (Wild) | Kadavu, Ravitaki  (Wild) | Savusavu,  Vatubukulaca  (Farm) | Savusavu,  Wailevu  (Farm) |
| --- | --- | --- | --- | --- | --- | --- | --- | --- | --- | --- |
| Raviravi  (Farm) | 0.0009 |  |  |  |  |  |  |  |  |  |
| Lau  (Wild) | 0.0050 | 0.0022 |  |  |  |  |  |  |  |  |
| Yasawa  (Wild) | 0.0126 | 0.0057 | 0.0026 |  |  |  |  |  |  |  |
| Udu Point  (Wild) | -0.0034 | -0.0045 | -0.0021 | -0.0015 |  |  |  |  |  |  |
| Taveuni  (Farm) | 0.0011 | -0.0010 | 0.0044 | 0.0109 | -0.0059 |  |  |  |  |  |
| Kadavu, Galoa  (Wild) | 0.0095 | -0.0001 | 0.0011 | -0.0123 | -0.0032 | 0.0137 |  |  |  |  |
| Kadavu, Ravitaki  (Wild) | 0.0072 | 0.0003 | -0.0039 | -0.0112 | -0.0063 | 0.0068 | -0.0142 |  |  |  |
| Savusavu, Vatubukulaca  (Farm) | 0.0050 | -0.0003 | 0.0014 | 0.0034 | -0.0027 | 0.0023 | 0.0019 | -0.0033 |  |  |
| Savusavu, Wailevu  (Farm) | 0.0025 | 0.0018 | 0.0104 | 0.0193 | -0.0019 | 0.0013 | 0.0173 | 0.0112 | 0.0093 |  |
| Savusavu, Wailevu  (Farm; hatchery) | 0.0967* | 0.0931* | 0.0908 | 0.1016 | 0.0850* | 0.0909 | 0.0932 | 0.0909 | 0.0873 | 0.0980* |

Significantly different values at p<0.000001 following 10,000 permutations are indicated with an asterisk.
